# Gender-sensitive school environment and bullying victimization among adolescent girls: A multilevel study in Nepal

**DOI:** 10.1371/journal.pone.0253128

**Published:** 2021-07-09

**Authors:** Irina Bergenfeld, Cari Jo Clark, Zara Khan, Emma C. Jackson, Kathryn M. Yount

**Affiliations:** 1 Department of Global Health, Rollins School of Public Health, Emory University, Atlanta, GA, United States of America; 2 Departments of Global Health and Sociology, Emory University, Atlanta, GA, United States of America; Washington University in St. Louis, UNITED STATES

## Abstract

**Background:**

Bullying is an understudied global social problem. While school-level factors are a recognized influence on bullying victimization, the elements of a ‘girl-friendly’ school that may reduce the risk of bullying victimization among girls and prevent dropout is understudied in lower- and middle-income countries (LMICs). This study used baseline data from the evaluation of the Room-to-Read (RtR) Girls’ Education Program (GEP) in Nepal to assess the relationship of a conceptually grounded gender-equitable school (GES) index with girls’ risk of direct and relational bullying victimization, adjusted for potential confounders at the individual and school levels.

**Methods:**

The school sample included all 24 RtR GEP schools and 25 community schools attended by girls in a comparison cohort, representing 729 grade six girls with complete outcome data. We employed multilevel negative binomial regression to assess the relationship between the GES score (higher scores indicate greater support for girls), and girls’ risk of peer victimization, controlling for individual- and school-level covariates.

**Results:**

On average, girls reported 2.84 direct victimizations and 0.27 relational victimizations in the prior week. The first component of the GES index, a generalized measure of school-level support for girls, showed a significant negative relationship with weekly relational bullying victimization in models with all school- and individual-level covariates. In the full model, a one-point higher score on the generalized GES component accounted for a 26% lower risk of relational bullying victimization in the prior week.

**Conclusion:**

School-level policies, practices, and pedagogy designed to support girls may reduce their exposure to relational aggression, a form of bullying that girls most often perpetrate. In LMICs, the school may be an ideal place to raise awareness about the types and effects of peer bullying and to promote prosocial bystander behavior. Further research is needed to identify factors related to other forms of bullying.

## Introduction

Bullying is a form of peer victimization with negative physical, mental, social and academic consequences for children and adolescents; yet, bullying remains an understudied problem in lower-income settings [1]. Available evidence from multi-country surveys suggests that experiences of bullying are widespread [1–5]; across 79 countries in 2003–2011, the estimated one to two month prevalence of bullying victimization in 11–15 year-olds was 32.4% for boys and 27.2% for girls, with widely varying national prevalence rates (from 12.4% in Armenia to 72.3% in Egypt for boys and from 8.7% in Italy to 46.3% in Zambia for girls) [6]. Estimates from UNICEF suggest comparable or higher prevalence rates [1, 2]; however, the types of violence included, exposure periods, and age-ranges of children vary across studies. Types of bullying victimization tend to vary by gender, with boys often at higher risk of physical victimization and girls often at higher risk of emotional, psychological, and relational victimization [7–15]. Youths who are differently abled, or who identify as part of ethnic or sexual minority groups, also are at higher risk of bullying victimization [16, 17].

In Nepal, 51% of 13-15-year-old respondents to the 2015 Global School-based Student Health Survey (GSHS) reported bullying victimization in the prior 30 days, with boys reporting higher overall exposure than girls (56% vs. 45%) [18]. About 40% of 13-15- year-olds reported not attending school at least one of the last 30 days because of feeling unsafe, and 70% of 13-15-year-olds reported sleeplessness due to worry over bullying victimization [18].

### Conceptualizing bullying

Bullying, as first defined, entails direct and indirect aggression that is intentional, repeated, and involving a physical or social imbalance of power, typically in school [19, 20]. In 2014, the Centers for Disease Control and Prevention (CDC) expanded this definition to entail “unwanted aggressive behavior(s) by another youth or group of youths who are not siblings or dating partners that involves an observed or perceived power imbalance and is repeated multiple times or is highly likely to be repeated. Bullying may inflict harm or distress on the targeted youth including physical, psychological, social, or educational harm” [21]. *Direct* aggression includes physical and verbal forms—such as hitting, shoving, or name-calling—that occurs in the presence of the target [21]. *Indirect* aggression entails exclusion, rumors, or gossip typically to decrease social status and occurs through a third party or when the target is not present [21]. In addition to *physical or verbal* aggression *and indirect or relational* aggression, other types of bullying include *damage to property* [21] and *cyberbullying*, or bullying through electronic forms of contact [22].

Among school-aged children and adolescents, these definitions of bullying have been criticized because of their de-emphasis of more serious, injurious, or aggravated forms of aggression, the difficulty of identifying and measuring power imbalances, and restriction to aggression in schools [23–25]. Questions of definition in this age group also have focused on the extent to which bullying overlaps with other forms of violence or victimization and the distinctions between forms of bullying [26]. Among those who have been bullied, the experience of multiple (for example, physical and verbal) forms of bullying is associated with poorer outcomes [27]. These results corroborate the broader literature on poly-victimization, which consistently finds that exposure to a greater number of adverse events is associated with worse psycho-social and health outcomes [28]. Thus, definitions of bullying should distinguish this form of violence from others (like dating violence) and should capture multiple types of bullying to improve estimates of prevalence and to capture gender-differentiated forms of bullying perpetration and victimization [29, 30].

Despite efforts to develop standard definitions for bullying in adolescents, the global literature reflects a range of terms and definitions. A common global definition may be difficult in practice, as descriptors and social mechanisms of bullying may differ cross-culturally [31–37]. Groups, such as UNESCO and UN Women, focus more broadly on child violence in schools, and include gender-based violence and sexual violence under this umbrella term [2, 3]. For this study, we follow the CDC’s definition of bullying, allowing for victimization to be during school hours, on the way to and from school, during school-related events, or during in-school or out-of-school tutoring [21].

### Academic and psychosocial effects of bullying

An emerging global body of evidence suggests a dose-response relationship between bullying victimization and academic and psychosocial outcomes. Experiences of bullying have been associated with lower scores for reading and math in 15 Latin American countries [16]. In a recent global systematic review and meta-analysis, the authors found that both traditional and cyber-bullying had strong relationships with a range of mental-health disorders, probable relationships with tobacco and illicit-drug use, and possible relationships with alcohol use, poor academic achievement, psychosomatic symptoms, social isolation, overweight and obesity [17]. In a meta-analysis of quasi-experimental studies in the Americas, United Kingdom, Ghana, Korea, and Italy, bullying victimization led to poorer short-term developmental outcomes, including higher internalizing and externalizing symptoms and reduced academic achievement [38]; however, these adverse effects appeared to diminish in the long-term, highlighting the potential for resilience in the victims of bullying. A smaller study among Virginia public-high-school students found that the perceived prevalence of teasing and bullying among ninth graders and teachers was positively associated with dropout rates four years later [39] and negatively associated with school-wide passing rates on state-mandated achievement tests [40, 41].

### The role of schools in a social-ecological model of girls’ bullying victimization

While gender parity in schooling has been achieved at primary level in Nepal, girls continue to lag behind boys in terms of overall attainment [42]. Lower secondary school, which begins at grade six, represents a time when girls’ risk of school dropout begins to increase, peaking at grade 9 [43]. The elements of a secondary school environment designed for improving educational and psychosocial outcomes for girls is unstudied in LMICs, though there is emerging evidence of an association between school-level gender equity and girls’ pass rates [44].

School-level factors are widely recognized to influence bullying victimization [45–47]; thus, schools may be an ideal entry point to address girls’ bullying victimization globally. While the elements of a favorable school climate continue to be debated, schools that foster the following practices may reduce peer victimization: bystander intervention; pro-social norms enforced by diligent monitoring; healthy, non-coercive relationships between teachers and students; and consistent communication of expectations between students, teachers, and families [48–57]. In randomized trials, school-based anti-violence programs have effectively reduced perpetration and victimization [58]; however, the effects of program components were not estimated, and rigorous, multi-armed intervention studies are lacking in low- and middle-income countries [59].

#### The gender and adolescence

Global Evidence (GAGE) conceptual framework is a useful one to understand the elements that may be needed to reduce the risk of bullying victimization among girls and assist them in achieving their full potential [60]. The GAGE framework emphasizes that girls exist within ecological systems that span their families, social networks, schools, communities, and societies. This framework also underscores that support for adolescent girls must be multidimensional within each domain of this social ecology. Schools are important not only to teach girls academic skills, but also to help girls develop the capacities for critical-thinking to function as adults. Schools provide girls with opportunities to socialize, to learn from role models, and to develop a wider view of the world. Because students are a “captive audience,” schools also can be a place where girls and boys encounter more gender equitable norms than those they encounter in other contexts. The United Nations Children Fund (UNICEF) criteria for a ‘child friendly’ school (CFS) also includes elements to ensure that schools are gender equitable and support the specific needs of girls [61]. Criteria include physical or social spaces that take girls’ needs into consideration; involvement of women and girls in decisions about school design; openness to girls’ perspectives and a willingness to foster gender-equitable norms; and dedication to the prevention of sexual violence.

Taking prior theory and the GAGE and UNICEF frameworks into account, a ‘girl-supportive’ school may include school policies that encourage retention in the context of early marriage, pregnancy, and childbearing; curricular instruction that supports healthy relationships, including sexual and reproductive health; school-based ‘reporting chains’ that alert authorities to planned illegal marriages; gender-segregated toilets to help girls address hygiene needs privately; the presence of female teachers; safe modes of transportation to and from school; and equal access to school materials and equal opportunities to participate in school-related activities. Therefore, a gender-sensitive environment includes *special support to girls* to overcome gender-specific barriers to schooling. Such support may include support from women teachers and staff who serve as role models, extra tutorial support to offset their more frequent absences, access to supportive, girl-only spaces, such as girls clubs, to enhance their voice, agency, and life skills, anonymous violence reporting boxes to enable girls (and boys) to report violence without fear of retribution, cash or in-kind transfers to girls to help them to continue their education, and school-based income-generation projects to allow girls to make the money they need to stay in school.

### Study objectives

This study used baseline data from the evaluation of the Room-to-Read (RtR) Girls’ Education Program (GEP) in Nepal to assess the relationship of a conceptually grounded gender-equitable school (GES) index [44] with girls’ risk of bullying victimization, adjusted for potential confounders at the individual and school levels.

## Methods

### Ethics

The Emory University Institutional Review Board [Approval#00103052] and the Nepal Health Research Council [Approval#237/2018] approved the study. Written informed consent was collected for all adult participants, and written parental consent and written assent for all minor participants. In accordance with GAGE and Emory University policies, field staff received training on additional human subjects considerations when conducting research among children and adolescents.

### Nepal setting

According to the 2016 Nepal Demographic and Health Survey, adolescents aged 10–19 years comprise 23% of the country’s population [42]. Girls in Nepal remain less likely than boys to complete secondary school, with increased risk of dropout beginning in early adolescence [43]. Barriers to girls’ adolescent capability development and associated social- and health-related challenges arise in part as a result of inequitable gender norms and entrenched social hierarchies that privilege men [62, 63]. Nepali girls and women still often are expected to be caretakers of the family and remain undervalued in society [64]. Practices like son preference, menstrual restrictions, dowry, child marriage, and polygamy persist and are symbolic of underlying gender-inequitable norms [62]. Girls and women also may experience unequal gender power relations, compromised voice and agency within their household and community, restrictions on their movement, and less access to formal schooling. Intimate partner violence (IPV) is an accepted norm among many men and women, and women’s exposure remains high [63].

### Study sites

The present study was undertaken in Tanahun and Nuwakot districts in Nepal. Both districts are characterized, overall, as having considerable socioeconomic disadvantage and low human development.

Nuwakot, located in the central region of Nepal, falls below the national average with respect to adult literacy (60% versus 66%), schooling attainment (3.3 versus 3.9 grades), and per capita income ($1,086 versus $1,160 PPP) [65]. Of the 12-year-old girls in Nuwakot in 2011, about 65% had finished primary school, 33% had finished lower secondary school, and 0.5% had completed secondary school [65]. The median age at first marriage in the province is 19.7 years among women 20–49 years [42]. That said, structural change is evident, with an influx of new schools, hospitals and improved roads in recent years [62].

In Tanahun district, per capita income ($1,072 PPP) falls below the national average, though average levels of schooling, 4.2 grades, are slightly higher [65]. Of the 12-year-old girls in Tanahun in 2011, an estimated 63% had finished primary school (Grades 1–5), 36% had finished lower secondary (Grades 6–8), and only 0.3% had completed secondary (Grades 9–10) [65]. In 2016–2017, the median age at first marriage across the province was 18.6 among women 20–49 years [42].

### Sample eligibility and response rates

The present analysis uses baseline data from the GAGE evaluation of the RtR GEP, which is underway in Nuwakot and Tanahun districts. We aimed to enroll approximately 580 grade six girls attending the 24 schools (12 per district) in which RtR was scheduled to roll out its GEP in 2018. GAGE provided the sampling frame for this cohort. We also aimed to establish a comparison cohort of 1,276 girls by taking a census of girls aged 12–14 years or in grade six in 40 randomly selected clusters (wards of <275 households or randomly selected segments of larger wards). Using the compact segment sampling method allowed us to produce a self-weighed sample with sufficiently high participation [66]. Eligible wards for the comparison cohort were adjacent to the wards in which the RtR GEP was being implemented and had a sufficient population of eligible girls. From this sampling frame, clusters were selected using probability proportional to size. The achieved sample at baseline included 571 girls in RtR GEP schools and a comparison sample of 1,127 girls. Baseline response rates were 99.8% of eligible RtR girls and 99.9% of eligible community girls, respectively.

All RtR GEP schools were visited and included in this analysis. Schools in the comparison clusters of Tanahun and Nuwakot districts were eligible for inclusion in this analysis if they were in the comparison area, were attended by a girl in the comparison sample, and were government schools, private schools, or government-supported public/community schools that had a grade six and above. For this analysis, we further restricted schools in the comparison clusters to those with greater than 40% of the reported female grade six enrollees sampled and excluded all schools with fewer than three girls sampled. The final school sample consisted of the 24 RtR GEP schools and 25 community schools, representing 750 girls in grade six. Twenty-one of the girls in this sample were missing outcome data and were removed, bringing the final sample to 729. Each girl attended one of 49 schools in the sample.

### Data collection

The field team included 30 female researchers who received a 13-day training undertaken jointly by GAGE team members at Emory University and the Centre for Research on Environment, Health, and Population Activities (CREHPA). The training included didactic sessions on field procedures, the survey questionnaires, methods for conducting the interview, GAGE policies, and ethics in human-subjects research. The field team received didactic and experiential training in the conduct of face-to-face computer-assisted personal interviews (CAPIs) using tablets and REDCap version 8.3.2. REDCap is a web-based data systems application developed by Vanderbilt University with funding from the National Institutes of Health that allows for the secure collection, storage, and transfer of multi-relational data and user-defined access by multi-institutional partners at any location in the world [67]. The training also included experiential role-plays, a pre-test, and a full pilot. The pre-test and pilot informed final revisions to the questionnaires and REDCap application. Fieldwork was undertaken from July 4, 2018 to September 2, 2018. After data collection, one debriefing session was held to give interviewers a platform to share feedback.

Adolescents were interviewed at home, in a private area where parents could see them but not overhear their answers. Interviews with adolescents took an average of 61 minutes. The school questionnaire, completed by a school administrator, consisted of 12 modules covering staff, policies, facilities and materials, student population, accessibility, support for students, and outside organizations operating within the school. School administrator interviews took place at the school building and lasted an average of 42 minutes. All questionnaires were developed in English, reviewed by RtR and the Overseas Development Institute, translated into Nepali, and back-translated into English to identify and resolve differences in the meaning and interpretation of the questions before piloting.

### Measures

To develop the questionnaire for this study, the study team relied on previously piloted and successfully implemented questionnaires from studies of adolescents in Nepal, studies of adolescents in the core Gender and Adolescent: Global Evidence consortium, and widely validated youth development scales (Table 1) [68]. Each question set that was included in the questionnaire was aligned with specific learning modules in the GEP program and was piloted and adapted for comprehension in the local setting and population.

**Table 1:** Survey modules and items collected.

Sample	Module	Variables collected (number)	Adapted from/Developed by	Example item
Adolescent	Demographics	Age	NA	NA
Adolescent	Early Educational Experiences	Age at first grade (1), financial assistance in primary school (1), attended preschool (1)	GAGE	During primary school, did you get any financial help in cash or kind from the school or another source?
Adolescent	Peer Networks	Peer gender-equitable attitudes (1), peer leadership (1)	Sociopolitical Control Scale for Youth [69]	[Peer] is often a leader in groups.
Adolescent	Health and Risky Behavior	Level of difficulty seeing, earing, speaking, moving, learning, and performing daily self-care tasks (6)	Washington Group Disability Questionnaire [70]	Please tell us whether you have no difficulty, some difficulty, a lot of difficulty, or are unable to walk or climb steps?
Adolescent	Aggression/Victimization	Direct aggression (4), relational aggression (2)	Reduced Aggression/Victimization Scale [71]	How many times in the last week has a classmate left you out on purpose?
Caregiver	Demographics	Education level (1)	NA	What is your highest completed class or grade?
Caregiver	Household Assets	Household socioeconomic status (10)^a^	Nepal 2010 Poverty Probability Index	What type of stove does your household mainly use for cooking?
School administrator	General characteristics	Type of school (1), grades taught (1)^b^, no. of teachers (1)^b^, no. of women teachers (1)^b^, teacher-student ratio (1)^b^, girls’ safety (1)^c^, repairs (1)^b^	GAGE	Can girls safely travel alone to and from school?
School administrator	Facility characteristics	Walls (1)^b^, roof (1)^b^, electricity (1)^b^, water (1)^b^, soap (1)^b^, toilets for girls (1)^c^, toilets for boys (1)^c^, unisex toilets (1)^c^, private spaces for menstrual management (1)^c^, computers (1)^b^, clubs for girls (1)^c^, co-ed clubs (1)^b^	GAGE	Does this school have drinking water? Does this school have private spaces, either in toilets or in a separate room, that girls can use during menstruation, for example to change clothes or pads?
School administrator	Gender demographics	Total enrollment of girls (1)^c^, no. girls dropped out (1)^c^, total enrollment of boys (1)^c^	GAGE	In the last school year, how many girls in total dropped out of school?
School administrator	Policies	Punishment types (1), policies keeping vulnerable girls in school (1)^c^, hours of SRH instruction (1)^c^	GAGE	What types of punishments are used at this school to correct a child’s misbehavior?

^a^Used as inputs in household socioeconomic status index;

^b^Used as inputs in school economic condition index;

^c^Used as inputs in GES index.

The adolescent questionnaire included 22 modules that covered the following domains: educational experiences in pre-kindergarten and primary school; positive youth development—including self-confidence, self-efficacy, positive self-identity, social competence, emotional competence, critical thinking, and leadership competence [72–75]; academic performance—including tests of reading comprehension and math skills [76]; marriage, job, and educational aspirations; voice, agency and freedom of movement; livelihoods, financial literacy, and economic empowerment; a safe and enabling environment; participation and civic engagement; adolescents’ social networks, reference groups, and role models; gender attitudes and norms—based on questions about masculinity, femininity, and gender relations from published scales [64, 77–79] experiences of school-based aggression and victimization [80]; health and risky behavior; and program benefits.

#### Outcome: Frequency of school bullying in the prior week

The six-item victimization sub-scale of the reduced aggression-victimization scale [71] was used to capture girls’ exposure to bullying in school. This sub-scale, developed for administration with 8–12 year-olds, has demonstrated high internal reliability in prior studies (Cronbach’s alpha 0.84) [71]. Four items capturing exposure to direct aggression ask, “How many times [DURING THE LAST SEVEN DAYS] did a kid from your school” “tease you,” “push, shove or hit you,” “call you a bad name,” or “say they were going to hit you.” Two items capturing exposure to indirect, relational victimization ask, “How many times [DURING THE LAST SEVEN DAYS] did a kid from your school” “leave you out on purpose” or “make up something about you to make other kids not like you anymore.” The response options for all six items range from 0 times to 6 or more times in the last seven days. Ranges for the victimization subscales are 0 to 24 for direct aggression and 0 to 12 for relational aggression. Cronbach’s alpha for the scale was adequate in this sample, at 0.68.

#### School-level exposure: Gender-equitable school index

An index for a gender sensitivity at the school-level [44], the main school-level exposure, was created using principal components analysis (PCA). The input variables from which components were derived operationalized the concept of a girl-supportive school, as conceptualized in the GAGE and UNICEF frameworks, described above. The final index included the availability of a room to change menstrual pads; presence of girls-only clubs; hours of sexual and reproductive health (SRH) instruction given beyond the required curriculum; administrator report that girls could travel safely to school; and policies to retain married, pregnant, and postpartum girls. With the exception of hours of SRH instruction, which was modeled continuously, each item was dichotomized to capture the presence (= 1) or absence (= 0) of that school characteristic. Additional constructed variables included in the index were the proportion of female students, proportion of female-only toilets, proportion of female teachers, and retention rates of female students. All variables were coded such that higher scores on the index indicated a more equitable school environment. Based on the scree plot showing proportion of variance explained, only the first two components retained in the gender equitable school (GES) index [81]. Component 1 was a generalized measure of girl-supportiveness that summarized several input variables; the variables with the highest loadings were the presence of girls-only clubs, proportion of female students, extra hours of SRH instruction, and retention policies for girls. Component 2 described girls’ safety and hygiene, with high (>0.35) loadings for whether girls could travel safely to school, proportion of female toilets, and the availability of a room to change menstrual pads.

#### Covariates

At the individual level, covariates included girls’ early educational experiences [82], household socioeconomic status (SES) [83], caregivers’ schooling level [84], and characteristics of peer networks [85]. Early educational experiences included whether girls attended preschool (dichotomous), whether girls received financial assistance in primary school (dichotomous), and girls’ reported age at first grade. A PCA score for household SES was calculated using the Nepal Poverty Probability Index and in accordance to DHS guidelines, with the first principal component retained [86]. Caregivers’ schooling level was the highest grade level the caregiver reported completing, analyzed as a continuous variable. Peer network characteristics were constructed from an egocentric peer network survey, which asked girls to list up to three individuals whom they would “trust to talk about something personal and private.” Participants were then asked to rate how much they agreed with two statements about these individuals using a four-point Likert scale: whether the individual was “often a leader in groups”, and whether the individual believed that “men and women should share the house work, such as doing dishes, cleaning and cooking.” Each item was then averaged over all peers to create measures of average peer leadership and average peer gender equitable attitudes, with higher scores indicating more agreement. We assessed disability using a summative scale derived from the six-item Washington Group Disability Questionnaire [70], which asks respondents to report whether they have no difficulty, some difficulty, a lot of difficulty, or cannot do certain activities.

At school level, covariates included a score for school economic condition (SEC) [87] constructed using PCA, the average students per grade level [88], school type (private vs. government/community) [89], school use of corporal punishment (dichotomous) [90], and urban/rural status of the school municipality [91]. Use of corporal punishment was determined by asking administrators to check all that applied from a list of disciplinary behaviors practiced at the school including corporal (whipping, hitting, caning, standing on a bench, etc.) and non-corporal (dismissal from class, etc.) forms of discipline. This measure was coded as 1 = yes if one or more corporal punishments were selected.

### Analysis

To assess the relationship between the gender-equitable school (GES) index, a contextual factor, and girls’ risk of peer victimization, we employed a multi-level approach [92]. First, we examined the distribution of all individual adolescent and school-level variables using frequencies, means, and standard deviations. Next, tetrachoric correlations among study variables were assessed separately by the level at which they were modeled (individual or school). As the outcome variable is a weighted count of victimization types that also is over-dispersed (scaled Pearson statistic different from 1), we used multilevel negative binomial regression for the estimation. We fit a series of models, the first of which was an unconditional random-intercept model (Model A) to estimate the amount of variability in victimization across schools. Subsequently, we fit a model containing the random intercept and the two GES components (Model B), followed by a model in which all school-level variables were also included (Model C). Finally, we estimated a full model (Model D), in which all individual- and school-level variables were modeled simultaneously. As the analytic focus was on the relationship of gender-sensitive school environment and victimization, we grand-mean-centered the individual-level variables so that the GES regression coefficients could be interpreted as the log count of victimization types, adjusted for potential confounding by individual variables [93]. Students per grade was grand-mean-centered for interpretability. We used PROC GLIMMIX in SAS (version 9.4, SAS Institute Inc., Cary, NC, USA) for the analysis.

## Results

### Characteristics of adolescent girls

Girls in the sample ranged in age from 9 to 17 years, with a mean age of 11.8 (Table 2). Approximately 40% had attended preschool. The mean age at starting first grade was 5.8 years, and 70% of girls reported having received financial assistance in primary school. On average, girls’ caregivers (mostly mothers) had received schooling through third grade. Mean household SES scores were approximately zero, with the lowest tertile ranging from negative 10 to negative 2 and the highest ranging from 1 to 8. On a scale of 1 to 4, average reported peer leadership was low, at 1.9, whereas average reported peer gender equity was high, at 3.6. On average, girls in our sample reported 3.12 victimizations in the prior week. Of these, 2.84 were direct victimizations and 0.27 were relational victimizations. The most frequently reported item was teasing, whereas the least commonly reported item was making up rumors. Girls from RtR schools were significantly less likely to have attended preschool and reported higher disability scores than their counterparts at community schools. They also reported higher levels of physical victimization in the prior week.

**Table 2:** Characteristics of adolescent girls (N = 729), Nuwakot and Tanahun districts, Nepal 2018.

	Overall (n = 729)		RTR sample (n = 570)		Community sample (n = 159)	
	Mean (SD)/n (%)	Range	Mean (SD)/n (%)	Range	Mean (SD)/n (%)	Range
Age at first grade	5.80 (1.21)	3–13	5.83 (0.05)	3–13	5.67 (0.09)	3–9
Attended Preschool*	281 (38.65%)	-	183 (32.22%)	-	98 (61.64%)	-
Financial assistance in primary	512 (70.23%)	-	393 (68.95%)	-	119 (74.84%)	-
Caregiver grades completed	3.32 (3.76)	0–14	3.24 (3.79)	0–14	3.62 (3.65)	0–14
Age	11.70 (1.24)	9–17	11.68 (1.26)	9–17	11.78 (1.16)	9–15
Household SES: Low	243 (33.33%)	-10.22–-1.8	193 (33.86%)	-	50 (31.45%)	-
Household SES: Medium	243 (33.33%)	-1.8–1.11	179 (31.40%)	-	64 (49.25%)	-
Household SES: High	243 (33.33%)	1.13–7.87	198 (34.74%)	-	45 (28.30%)	-
Average peer leadership	1.90 (0.99)	1–4	1.91 (0.99)	1–4	1.85 (0.54)	1–4
Average peer gender equitable attitudes	3.65 (0.54)	1–4	3.64 (0.54)	1–4	3.69 (0.52)	1–4
Disability score*	6.53 (0.99)	6–14	6.59 (1.03)	6–14	6.33 (0.76)	6–12
**Times in the last week a classmate…**						
…teased you	1.31 (1.81)	0–6	1.37 (1.86)	0–6	1.11 (1.63)	0–6
…pushed, shoved, or hit you*	0.55 (1.18)	0–6	0.61 (1.26)	0–6	0.40 (0.83)	0–4
…called you a bad name	0.66 (1.42)	0–6	0.68 (1.45)	0–6	0.58 (1.29)	0–6
….said that they were going to hit you	0.31 (0.97)	0–6	0.34 (0.99)	0–6	0.24 (0.88)	0–5
Direct victimization summative score*	2.84 (4.01)	0–30	3.00 (4.08)	0–24	2.28 (3.71)	0–22
…left you out on purpose	0.14 (0.53)	0–5	0.11 (0.49)	0–3	0.13 (0.57)	0–6
…made up something about you…	0.13 (0.58)	0–6	0.15 (0.62)	0–6	0.08 (0.40)	0–3
Relational victimization summative score	0.27 (0.89)	0–8	0.29 (0.93)	0–8	0.20 (0.72)	0–5
Total victimization summative score*	3.12 (4.34)	0–30	3.29 (4.41)	0–30	2.47 (4.03)	0–24

*significant difference between arms at p<0.05.

### Characteristics of schools

The overwhelming majority of sampled schools were government or community schools, with fewer than five percent private (Table 3). About two thirds were in urban municipalities, and located in Tanahun or adjacent areas of Gorhka and Lamjung districts. A large majority of schools did not report using corporal punishment as a means of discipline. On average, schools enrolled 37 students per grade, although this number ranged from about 8 to almost 150. SEC ranged from -3.89 to 2.31, with mean of zero. Mean GES scores also were approximately zero, ranging from -3.03 to 4.97 for component 1 and -3.42 and 2.78 for component 2. RtR schools were significantly more likely to be in Nuwakot and public. In addition, RtR schools were more likely to have a higher economic condition, had more students per grade, and had higher average reported disability scores.

**Table 3:** Characteristics of schools, community sample (N = 25) and RtR school sample (N = 24), Nuwakot and Tanaun districts, Nepal 2018.

	Overall (n = 49)		RtR Schools (n = 24)		Community (n = 25)	
School-level variables	Mean (SD)/%	Range	Mean (SD)/%	Range	Mean (SD)/%	Range
Nuwakot (vs. Tanahun)*	32.65%	-	50.00%	-	16.00%	-
Public (vs. private)*	85.71%	-	100%	-	72.00%	-
Urban (vs. rural)	65.31%	-	66.67%	-	64.00%	-
Does not use corporal punishment	87.76%	-	87.50%	-	88.00%	-
School Economic Condition: Low*	34.69%	-3.89–-0.69	16.67%	-	52.00%	-
School Economic Condition: Medium*	32.65%	-0.66–0.72	33.33%	-	32.00%	-
School Economic Condition: High*	32.65%	0.74–2.11	50.00%	-	16.00%	-
Students per grade*	34.77 (23.20)	7.88–149.25	46.62 (26.24)	22.33–149.25	23.39 (11.90)	7.89–53.4
GES General: Low	34.69%	-1.83–-0.20	29.17%	-	40.00%	-
GES General: Med	32.65%	-0.15–1.25	37.50%	-	28.00%	-
GES General: High	32.65%	1.46–7.76	33.33%	-	32.00%	-
GES Safety and Hygiene: Low	34.69%	-1.47–-0.09	29.17%	-	40.00%	-
GES Safety and Hygiene: Med	32.65%	-0.07–0.95	29.17%	-	36.00%	-
GES Safety and Hygiene: High	32.65%	0.98–4.97	41.67%	-	24.00%	-
Aggregated individual-level variables						
Age at first grade	5.86 (0.54)	4.70–6.86	5.86 (0.44)	5.05–6.62	5.71 (0.62)	4.70–6.86
Attended Preschool	38.64%	-	32.22%	-	61.64%	-
Financial assistance in primary school	70.23%	-	85.32%	-	79.87%	-
Caregiver grades completed	3.21 (2.22)	0.00–10.00	2.82 (1.60)	0.76–7.43	3.58 (2.66)	0.00–10.00
Age	11.76 (0.71)	10.20–13.67	11.73 (0.47)	10.84–12.89	11.76 (0.71)	10.2–13.67
Household SES tertile (score range)	2.00 (0.40)	1.00–3.00	1.98 (0.25)	-2.46–1.05	2.02 (0.51)	-3.23–3.36
Average peer leadership	1.90 (0.46)	1.00–3.67	1.88 (0.33)	1.31–2.48	1.92 (0.57)	1.00–3.67
Avg. peer gender equitable attitudes	3.65 (0.24)	3.00–4.00	3.63 (0.16)	3.36–4.00	3.68 (0.30)	3.00–4.00
Disability score*	6.44 (0.38)	6.00–7.50	6.61 (0.34)	6.13–7.50	6.28 (0.34)	6.00–7.40
Direct victimization score	2.61 (1.31)	0.60–6.19	2.95 (1.32)	0.81–6.19	2.29 (1.24)	0.60–5.38
Relational victimization score	0.23 (0.29)	0.00–1.50	0.25 (0.26)	0.00–0.10	0.21 (0.33)	0.00–1.50
Total victimization score	2.85 (1.51)	0.68–7.29	3.20 (1.52)	0.92–7.29	2.50 (1.45)	0.68–6.88

*significant difference between arms at p<0.05.

### School environment and bullying victimization

In multilevel models, no school-level covariates were significantly associated with weekly rate of either total bullying victimization or direct bullying victimization, although caregiver education was negatively associated with both direct and total bullying victimization, and higher levels of disability were positively associated with total victimization (Table 4). The generalized GES component showed a significant negative relationship with weekly relational bullying victimization in Models C (with all school-level covariates) and D (with all school- and individual-level covariates). In the full model, a one-point increase in the generalized GES was associated with a 26% decrease in relational bullying victimization in the prior week. In full models, attending a government or community school also was positively associated with the weekly victimization rate of relational bullying, accounting for a 16-fold increase versus attending a private school.

**Table 4:** Multilevel models of gender equitable school-level exposures and total victimization, N = 729 adolescent girls in Nuwakot and Tanahun districts in Nepal, 2018.

**Direct victimization**	**Model A**			**Model B**			**Model C**			**Model D**		
**Effect**	**Est.**	**95% CI**		**Est.**	**95% CI**		**Est.**	**95% CI**		**Est.**	**95% CI**	
**Random effects variance**	0.92**	0.72	1.12	0.95**	0.72	1.18	0.61	-0.28	1.51	0.51	-0.37	1.39
**GES General**				-0.04	-0.15	0.08	-0.05	-0.17	0.07	-0.06	-0.18	0.05
**GES Safety and Hygiene**				0.02	-0.16	0.19	0.02	-0.17	0.20	0.01	-0.17	0.19
**School Economic Condition**							-0.10	-0.25	0.05	-0.11	-0.25	0.04
**Students per grade**							0.00	0.00	0.01	0.00	0.00	0.01
**School type (ref. private)**							0.38	-0.36	1.12	0.50	-0.22	1.23
**No corporal punishment**							-0.06	-0.65	0.53	-0.11	-0.69	0.47
**Urban**							0.08	-0.33	0.49	0.11	-0.29	0.51
**Nuwakot**							0.12	-0.31	0.54	0.18	-0.24	0.59
**Attended Preschool**										0.17	-0.12	0.46
**Age at grade 1**										-0.01	-0.12	0.10
**Financial aid in primary**										0.08	-0.20	0.37
**Household SES**										0.01	-0.03	0.05
**Caregiver grades completed**										-0.05**	-0.09	-0.02
**Average peer leadership**										0.11	-0.01	0.24
**Avg. peer gender attitudes**										-0.03	-0.26	0.20
**Disability score**										0.12	-0.01	0.24
**Relational victimization**	**Model A**			**Model B**			**Model C**			**Model D**		
**Effect**	**Est.**	**95% CI**		**Est.**	**95% CI**		**Est.**	**95% CI**		**Est.**	**95% CI**	
**Random effects variance**	-1.57**	-2.06	-1.09	-1.29**	-1.86	-0.72	-3.71**	-6.20	-1.22	-3.99**	-6.52	-1.46
**GES General**				-0.13	-0.35	0.09	-0.20*	-0.38	-0.02	-0.30**	-0.49	-0.10
**GES Safety and Hygiene**				-0.11	-0.42	0.20	-0.07	-0.37	0.22	-0.08	-0.37	0.21
**School Economic Condition**							-0.11	-0.38	0.15	-0.05	-0.33	0.22
**Students per grade**							0.00	-0.01	0.01	0.00	-0.01	0.01
**School type (ref. private)**							2.55*	0.28	4.82	2.75*	0.47	5.03
**No corporal punishment**							0.26	-0.67	1.20	0.23	-0.72	1.18
**Urban**							-0.08	-0.67	0.51	0.10	-0.53	0.73
**Nuwakot**							-0.22	-0.87	0.43	-0.23	-0.88	0.42
**Attended Preschool**										-0.12	-0.84	0.59
**Age at grade 1**										0.22	-0.03	0.48
**Financial aid in primary**										0.14	-0.58	0.86
**Household SES**										-0.04	-0.13	0.06
**Caregiver grades completed**										-0.02	-0.12	0.07
**Average peer leadership**										-0.10	-0.38	0.19
**Avg. peer gender attitudes**										-0.07	-0.60	0.46
**Disability score**										0.25	-0.03	0.53
**Total victimization**	**Model A**			**Model B**			**Model C**			**Model D**		
**Effect**	**Est.**	**95% CI**		**Est.**	**95% CI**		**Est.**	**95% CI**		**Est.**	**95% CI**	
**Random effects variance**	0.98**	0.77	1.19	1.01**	0.77	1.25	0.60	-0.33	1.53	0.51	-0.39	1.41
**GES General**				-0.04	-0.16	0.09	-0.06	-0.18	0.07	-0.07	-0.19	0.05
**GES Safety and Hygiene**				0.00	-0.18	0.18	0.00	-0.19	0.19	0.00	-0.19	0.18
**School Economic Condition**							-0.11	-0.26	0.05	-0.11	-0.26	0.05
**Students per grade**							0.00	0.00	0.01	0.00	0.00	0.01
**School type (ref. private)**							0.49	-0.27	1.25	0.61	-0.13	1.35
**No corporal punishment**							-0.08	-0.70	0.53	-0.13	-0.73	0.47
**Urban**							0.10	-0.33	0.54	0.13	-0.29	0.55
**Nuwakot**							0.10	-0.34	0.55	0.16	-0.27	0.59
**Attended Preschool**										0.14	-0.15	0.43
**Age at grade 1**										0.01	-0.10	0.12
**Financial aid in primary**										0.06	-0.22	0.34
**Household SES**										0.01	-0.03	0.04
**Caregiver grades completed**										-0.05**	-0.09	-0.01
**Average peer leadership**										0.09	-0.03	0.22
**Avg. peer gender attitudes**										-0.03	-0.26	0.19
**Disability score**										0.13*	0.01	0.25

Est. = estimate. CI = Confidence Interval. Students per grade was grand mean centered at the cluster level.

*p-value <0.05;

**p-value <0.01.

## Discussion

To our knowledge, this is the first study to examine the relationship between the gender-equitable school climate, as measured by indices our team has developed [44], and experiences of bullying among adolescent girls in LMICs. Overall, only individual-level covariates, namely disability and caregiver education, were significantly associated with experiences of total bullying victimization in our sample. However, the first component of the GES index, which had high loadings for girls-only clubs, retention policies for vulnerable girls, proportion of female students, and extra SRH instruction, was negatively associated with experiences of relational bullying victimization in the prior week in models incorporating school-level covariates, as well as in models with both individual- and school-level covariates. In the same models, attending a government or community school was associated with increased risk of relational bullying victimization in the prior week. Overall, this suggests that the school environment may be an influential determinant in peer bullying victimization and a promising target for interventions, and that fostering a school environment that is explicitly supportive of girls may mitigate other risk factors for relational bullying, such as attending a lower-resourced school. Further research is needed to identify environmental factors that may influence direct forms of bullying.

This analysis is aligned with international research suggesting that relational aggression is a gendered form of bullying that is more relevant to girls [31, 94, 95]. In studies conducted among middle-school students in the US, bullying tended to be same-gender, with boys primarily bullying boys and girls primarily bullying girls [96]. Same-gender bullying may be especially relevant in environments where adolescent interactions with their peers are primarily homosocial and social interaction between boys and girls is discouraged, as is the case in Nepal [97]. Bullying, and especially relational aggression, has been posited as a way in which girls “act out” in environments where they have relatively little power [98], as well as a means by which girls “perform gender” by reaffirming hegemonic femininity through social exclusion of female peers who violate feminine norms [94]. In environments where girls feel more supported, they may be less likely to perpetrate social aggression against peers; additionally, girls may be feel more empowered to intervene against bullying in more girl-supportive school environments. Having safe spaces to interact with other girls after school may be a primary driver of this impact.

With respect to other covariates, such as peer-relationships, research has shown that youths who are part of positive peer friendships and social networks have a lower risk of peer victimization [49, 99–102]. Although we did not find a significant association with peer characteristics and the rate of bullying victimization in our sample, understanding the direct associations of school climate and peer networks is important in future research. Other characteristics of peer networks, such as friendship quality and mutual support, are worth considering as potential factors influencing adolescent girls’ risk of bullying victimization [100]. Finally, our analysis confirms prior research identifying disability and socioeconomic status as risk factor for bullying victimization [16, 17]. When designing school environments that support girls, it is crucial to consider the ways in which gender intersects with other factors that can influence students’ risk of dropping out of school.

### Strengths and limitations

The scope of this study, which considered individual, household, peer-network, and school-level factors, allowed the research team to better understand how the nested environments in which adolescent girls operate may impact their risk of peer victimization. While direct victimization in the prior week was more commonly reported than relational victimization, the scale measure used included more items to measure direct aggression. Almost 90% of girls in our sample reported having some access to cell phones, and more than a third of these could access the internet. As cell phone and internet access expand, increasing opportunities for cyberbullying, accurately measuring relational aggression may require new items to be added to existing, validated scales. Additionally, we did not ask girls for the gender or other characteristics of perpetrators, limiting our ability to draw conclusions about the relative prevalence of same-gender versus cross-gender bullying and the relative prevalence of different forms of aggression perpetrated by girls versus by boys. Finally, under GAGE’s ethics policies, we were not able to collect individual-level data on experiences of violence perpetrated by adults. Measures of corporal punishment based on administrator report may be underestimates due to the illegality of the behavior [103].

## Conclusions

Our study suggests that school-level girl-supportive policies, practices, and pedagogy may reduce girls’ risk of experiencing relational aggression, a form of bullying most often perpetrated by other girls. In lower-income settings like our study districts, the school may, therefore, be an ideal setting to address peer victimization through interventions that raise awareness about the types and harmful effects of bullying and promote prosocial behavior. Further research is needed to identify factors related to other forms of bullying.

## Supporting information

S1 File SAS output. (TXT)
